# Glucose-Sensitive Holographic (Bio)Sensors: Fundamentals and Applications

**Published:** 2017-11

**Authors:** Marjan Heidari, Bahareh Dabirmanesh, Hossein Mahdavi, Khosro Khajeh

**Affiliations:** 1 Department of Biochemistry, Faculty of Biological Sciences, Tarbiat Modares University, Tehran, Iran; 2 School of Chemistry, Faculty of Sciences, University of Tehran, Tehran, Iran

Nowadays sensors and especially biosensors play an important role in medical diagnosis and detection of food and environment contaminations. Biosensors’ facilities have been improved significantly by using technologies such as surface plasmon resonance, microfluidics, laser, and electrochemistry. These technologies are now available on chips in micro- and nano-scale and are capable of measuring the quantity of more than one analyte at once. One branch of these sensors is the holographic (bio)sensor. Reflection holographic sensors, which are optical devices fabricated based on Bragg’s law, can diffract light with specific wavelength as they absorb or release special ions or molecule depending on their polymer structure. Their action is reversible and rapid and by measuring the color change, it is possible to determine the concentrations in real-time. Reversibility let the operator use a sensor several times. There are many sensors designed for measuring ethanol, metal ions, enzymes activity, and glucose.

In 2012, WHO announced that diabetes was the 8^th^ major cause of death worldwide. In 2014, the International Diabetes Foundation estimated that diabetes was directly or indirectly the cause of 4.9 million deaths in the world. Therefore, glucose quantification is very important and has applications in screening and medical diagnostics. Nowadays, the usual methods for evaluating the levels of glucose in body fluids are urine strip tests and glucose meters. The main problems with these approaches are enzymes purification during sensors fabrication and their inactivation after production. Also in some methods, the results are not highly accurate and reliable.

Here, we introduce a new glucose sensor and describe its benefits over other sensors, in design and fabrication. This optical sensor reaction toward glucose is based on Bragg’s law. Accordingly, if the space between the layers of the polymer matrix of the sensor can be changed in the presence of glucose, the ultimate wavelength of the diffracted light will be different from the wavelength of the incident light. Hologram sensors are reflective devices consisting of a polymer matrix with distributed nanoparticles between its layers, which can diffract light with high specificity of glucose intake to the polymer. Also, the change in its color is reversible, and it can gain its prior color after releasing glucose whilst each strip of enzyme-based sensors can be used only once. Polymer matrix in these holograms consists of polyacrylamide matrix with functional groups of boronic acid derivatives. *Cis* diols such as glucose can attach to 3-APB or 3-(Acrylamido)phenylboronic acid and form covalent bonds. Boronic acid with pK_a_=8.8 is stable at the pH lower than 8.8 in a trigonal planar configuration. Absorbing glucose molecules by the polymer matrix causes boronic acid-glucose complexes formation with lower pK_a_ and negatively charged tetrahedral structures. Hence at physiological pH, the charged groups of these complexes cause an increase in Donnan osmotic pressure, and afterwards, the polymer matrix can intake water and swell. Swelling of the hydrogel results in elevating the space between the polymer layers and nanoparticles, respectively. According to Bragg’s law, when the space between polymer layers is changed, a radiation with specific wavelength will be diffracted. This phenomenon occurs due to Ag° nanoparticles inside the polymer, which act as tiny mirrors, with longer wavelength that causes a change in the color of the sensor; which is actually a pseudo color. After washing and releasing the agents, layers of polymer will return to their initial places, and the sensor gains its original color. This reversibility lets this sensors be used several times.

Using this technology to fabricate contact lenses was a great step in diagnostics. Contact lenses act as minimally invasive sensors to measure concentrations of glucose and some other analytes in tear. As the procedure of sensing glucose was mentioned above, the contact lenses can absorb glucose and response to different glucose concentrations with different colors. Unlike traditional methods, this approach can provide a fast and an accurate diagnosis for consumers without causing any pain or bleeding.

**More details in:**

***A novel enzyme based SPR-biosensor to detect bromo-criptine as an ergoline derivative drug***. S Jabbari *et al*. 2017. Sens Actua B Chem, Vol. 240: pp. 519-527.

***Development of a label-free SPR sensor for detection of matrix metalloproteinase-9 by antibody immobilization on carboxymethyldextran chip***. S Mohseni *et al*. 2016. Biosens Bioelectron, Vol. 81: pp. 510-516.

***Fundamentals of holographic sensing***. In: Holographic Sensors (pp. 27-51). AK Yetisen 2015. Springer International Publishing.

***Holographic glucose sensors***. S Kabilan *et al*. 2005. Biosens Bioelectron, Vol. 20: 1602-1610.

